# ﻿Outline, phylogenetic and divergence times analyses of the genus *Haploporus* (Polyporales, Basidiomycota): two new species are proposed

**DOI:** 10.3897/mycokeys.98.105684

**Published:** 2023-07-24

**Authors:** Heng Zhao, Josef Vlasák, Yuan Yuan

**Affiliations:** 1 Institute of Microbiology, School of Ecology and Nature Conservation, Beijing Forestry University, Beijing 100083, China Beijing Forestry University Beijing China; 2 Biology Centre of the Academy of Sciences of the Czech Republic, Branišovská 31, CZ-370 05 České Budějovice, Czech Republic Biology Centre of the Czech Academy of Sciences České Budějovice Czech Republic

**Keywords:** fungal diversity, new taxa, molecular clock dating, wood-inhabiting fungi

## Abstract

*Haploporus* species have a worldwide distribution and 27 species have been accepted. In this study, two new species, *Haploporuscrystallinus* and *H.dextrinoideus*, are proposed from South America, based on the molecular fragments (ITS, LSU and mtSSU) and morphological evidence. Molecular clock analysis was performed and the result suggests that the ancestor of Polyporales originated between the Late Jurassic and Early Cretaceous period, with a mean stem of 159.8 Mya [95% higher posterior density (HPD) of 142.4–184.1 Mya] and the genus *Haploporus* occurred at a mean stem of 108.3 Mya (95% HPD of 88.5–128.2 Mya). In addition, most species of the genus are diversified between 60.5 Mya and 1.8 Mya, during the Paleogene to Neogene. A key to the accepted species of the genus *Haploporus* is provided.

## ﻿Introduction

Currently, more than 155,755 species of fungi have been described worldwide, based on molecular analyses and morphological features and the numbers of fungal species have been rapidly increasing in the last two decades (https://www.speciesfungorum.org/Names/Names.asp, accessed 21 June 2023). For example, Kirk et al. (2008) recorded 487 species of the family Hymenochaetaceae in the 10^th^ version of Dictionary of the Fungi, but 672 poroid species in 34 genera were recognised in 2022 (Wu et al. 2022a). Still, the fungal diversity is poorly known as only 4.10–7.08% fungal species have been described of an estimated 2,200,000–3,800,000 (Hawksworth and Lücking 2017; Wang et al. 2019).

The genus *Haploporus* Bondartsev & Singer, proposed by A. S. Bondartsev and R. Singer and typified by *Haploporusodorus* (Sommerf.) Bondartsev & Singer (Singer 1944), belongs to Polyporaceae, Polyporales, Agaricomycetes in Basidiomycota. The genus is characterised by: 1) basidiomata annual to perennial and resupinate to pileate; 2) hyphal system monomitic, dimitic to trimitic with clamped generative hyphae; 3) cyanophilous skeletal hyphae; 4) cyanophilous and ornamented basidiospores; and 5) causing a white rot (Singer 1944; Dai et al. 2002; Piątek 2003, 2005; Li et al. 2007; Shen et al. 2016; Zhou et al. 2019, 2021; Decock et al. 2021; Man et al. 2023).

A phylogenetic study suggested that the genus *Haploporus* was sister to the genus *Perenniporia*, based on the ITS + LSU + mtSSU + rpb2 + TEF1 sequences and 13 species have been accepted (Shen et al. 2016). Later, ITS + LSU and ITS + LSU + mtSSU sequences were used to reconstruct the phylogenetic relationships of the genus *Haploporus*, respectively (Zhou et al. 2019, 2021; Decock et al. 2021; Man et al. 2023), the results showing that they were compatible.

Until now, 27 species are accepted in the genus *Haploporus*, widely distributed in Africa, Asia, Europe, North America, Oceania and South America (Wu et al. 2022b; Man et al. 2023). In Africa, four species, *H.eichelbaumii*, *H.grandisporus*, *H.nanosporus* and *H.papyraceus*, are described (Decock et al. 2021). In Asia, 16 species are reported, accounting for 59.3% (16/27) of total species of the genus and are mainly found in China and Sri Lanka (Dai et al. 2004, 2007, 2021; Li et al. 2007; Dai 2012a, b; Shen et al. 2016; Zhou et al. 2019, 2021; Man et al. 2023). In Europe, three species are accepted, viz. *H.odorus*, *H.subtrameteus* and *H.tuberculosus* (Ryvarden and Gilbertson 1993; Zhou et al. 2019). In North America, four species are reported, including *H.alabamae*, *H.gilbertsonii*, *H.odorus* and *H.papyraceus* (Zhou et al. 2019). In Oceania, only one species, *H.pirongia*, is found from Australia and New Zealand (Zhou et al. 2019). In South America, four species are recorded from Brazil and Ecuador (Lira et al. 2018; Zhou et al. 2021; Man et al. 2023). Most species of the genus *Haploporus* grow on angiosperms, including branches, twigs, fallen trunks, stump, even living trees, while *H.latisporus* is the sole species growing on gymnosperm wood (Li et al. 2007; Zhou et al. 2019, 2021).

Molecular clock analyses provided new insights into the origin and evolution of fungi, such as reconstructing the divergence time of Basidiomycota and early-diverging fungi, based on multiple gene loci (Cao et al. 2012; Wu et al. 2014; Chen et al. 2015; Cui et al. 2018; He et al. 2019; Varga et al. 2019; Zhao et al. 2022a). The previous study suggested that divergence times of the order Polyporales, the genus *Polyporus* and its allied genera, originated about 141.81 Mya and 49–63 Mya years ago, based on six DNA fragments (Ji et al. 2022). The divergence times of *Haploporus* species have not yet been studied.

During the trips in South America, we collected some specimens of the genus *Haploporus* and carried out detailed studies combining morphology, phylogeny and molecular clock dating. Thus, two new species, *Haploporuscrystallinus* and *H.dextrinoideus*, are described in this study.

## ﻿Materials and methods

### ﻿Morphological studies

In the present study, the newly-studied specimens of the genus *Haploporus* were collected from South America, deposited in the herbarium of the Institute of Microbiology, Beijing Forestry University (**BJFC**, China), the National Museum Prague of Czech Republic (**PRM**, Czech Republic) and the private herbarium of Josef Vlasák (**JV**, Czech Republic). The methods of morphological description followed the previous study (Dai 2010; Chen et al. 2016). L = mean spore length (arithmetic average of all spores), W = mean spore width (arithmetic average of all spores), Q = variation in the L/W ratios between the specimens studied, n = x/y = number of spores (x) measured from a given number (y) of specimens (Yuan et al. 2017). The colour terms used by Anonymous and Petersen are followed (Anonymous 1969; Petersen 1996).

### ﻿DNA extraction, PCR amplification and sequencing

Total genomic DNAs were extracted from dried specimens using a kit (Aidlab Biotechnologies, Beijing, China), following the manufacturer’s instructions. Polymerase chain reaction (PCR) was used to amplify the partial fragments of the internal transcribed spacer (ITS), large subunit ribosomal RNA (LSU) and small subunit mitochondrial rRNA gene (mtSSU) with the fungal-specific primers, according to the previous studies (Zhou et al. 2019, 2021; Man et al. 2023). The amplification of the ITS, LSU and mtSSU partial fragments were carried out under the following conditions: for ITS and mtSSU partial fragments, an initial denaturation at 95 °C for 3 min, followed by 34 cycles at 94 °C for 40 s, 54 °C for 45 s and 72 °C for 1 min and a final extension of 72 °C for 10 min; for LSU partial fragments, an initial denaturation at 94 °C for 1 min, followed by 34 cycles at 94 °C for 30 s, 50 °C for 1 min and 72 °C for 1.5 min and a final extension of 72 °C for 10 min (Zhou et al. 2019, 2021; Man et al. 2023). Sequencing was performed by BGI Tech Solutions (Beijing Liuhe Co., Ltd., Beijing, China) using the ABI-3730-XL DNA Analyzer (Applied Biosystems, Foster City, CA, USA). All the newly-generated sequences are deposited in GenBank database with the accession numbers listed in Table 1.

**Table 1.** Taxa information and GenBank accession numbers used in this study.

Species	Sample no.	GenBank Accession no.			Country	References
		ITS	LSU	mt-SSU		
*Haploporusalabamae*	Dollinger 895	KY264038	MK433606	MW463004	USA	Zhou et al. (2019)
*H.alabamae*	JV 1704/75	MK429754	MK433607	MW463005	Costa Rica	Zhou et al. (2019)
*H.angustisporus*	Dai 10951	KX900634	KX900681	MW463006	China	Zhou et al. (2019)
*H.bicolor*	Dai 19951	MW465684	MW462995	–	China	Zhou et al. (2021)
*H.crassus*	Dai 13580	MW465669	KU941865	–	China	Zhou et al. (2019)
***H.crystallinus***	**JV 2208/36**	**OQ919235**	**OQ919238**	**OQ919241**	**French Guiana**	**This study**
	**FG-14-870**	MT782653	MT777443	–	**French Guiana**	Decock et al. (2021)
*H.cylindrosporus*	Dai 15664	KU941854	KU941878	KU941903	China	Shen et al. (2016)
***H.dextrinoideus***	**JV 2211/1-J**	**OQ919237**	**OQ919240**	**OQ919242**	**Ecuador**	**This study**
	**JV 2106/45-J**	**OQ919236**	**OQ919239**	–	**Ecuador**	**This study**
*H.ecuadorensis*	JV1906/C10-J	MW465661	OP948227	OP948226	Ecuador	Man et al. (2023)
*H.eichelbaumii*	Congo 1	MT758256	MT758256	–	Congo	Decock et al. (2021)
	KE-17-238	MT758261	MT758261	–	Kenya	Decock et al. (2021)
*H.gilbertsonii*	JV 1611/5-J	MK429756	MK433609	MW463007	USA	Zhou et al. (2019)
*H.grandisporus*	KE-16-130	MT758242	MT758242	–	Kenya	Decock et al. (2021)
	KE-17-228	MT758244	MT758244	–	Kenya	Decock et al. (2021)
*H.latisporus*	Dai 11873	KU941847	KU941871	MW463008	China	Shen et al. (2016)
*H.longisporus*	JV 1906/C11-J	MW465685	MW462996	–	Ecuador	Zhou et al. (2021)
*H.microsporus*	Dai 12147	KU941861	KU941885	–	China	Shen et al. (2016)
*H.monomitica*	Dai 24229	OP725709	OP725712	–	China	Man et al. (2023)
	Dai 24246	OP725710	OP725713	OP725715	China	Man et al. (2023)
	Dai 24251	OP725711	OP725714	OP725716	China	Man et al. (2023)
*H.nanosporus*	MUCL 47447	MT782648	MT777438	–	Gabon	Shen et al. (2016)
	MUCL 47559	MT782650	MT777440	–	Gabon	Shen et al. (2016)
*H.nepalensis*	Dai 12937	KU941855	KU941879	KU941904	China	Shen et al. (2016)
*H.odorus*	Dai 11296	KU941845	KU941869	KU941894	China	Shen et al. (2016)
	Yuan 2365	KU941846	KU941870	KU941895	China	Shen et al. (2016)
*H.papyraceus*	Dai 10778	KU941839	KU941863	KU941888	China	Shen et al. (2016)
*H.pirongia*	Dai 18659	MH631017	MH631021	MW463009	Australia	Zhou et al. (2019)
*H.punctatus*	Dai19628	MW465687	MW462998	MW463011	Sri Lanka	Zhou et al. (2021)
*H.septatus*	Cui 4100	KU941844	KU941868	KU941893	China	Shen et al. (2016)
*H.srilankensis*	Dai19523	MW465688	MW462999	MW463012	Sri Lanka	Zhou et al. (2021)
*H.subpapyraceus*	Cui 2651	KU941842	KU941866	KU941891	China	Shen et al. (2016)
	Dai 9324	KU941841	KU941865	KU941890	China	Shen et al. (2016)
*H.subtrameteus*	KUC20121102-36	KJ668536	KJ668389	–	Korea	Shen et al. (2016)
*Haploporus* sp. 1	LR11231	MT758249	MT758249	–	Malawi	Decock et al. (2021)
*H.thindii*	Cui 9373	KU941851	KU941875	KU941900	China	Shen et al. (2016)
	Cui 9682	KU941852	KU941876	KU941901	China	Shen et al. (2016)
*H.tuberculosus*	15559	KU941857	KU941881	KU941906	Sweden	Shen et al. (2016)
*Perenniporiacitrinoalba*	Dai 13643	NR_171808	NG_075212	KX880705	China	Cui et al. (2019)
*P.hainaniana*	Cui 6364	JQ861743	JQ861759	KF051044	China	Zhao and Cui (2013)

Note: The sequences generated in this study are in bold. “−” represents sequences unavailable.

### ﻿Phylogenetic analyses

**Figure 1.**
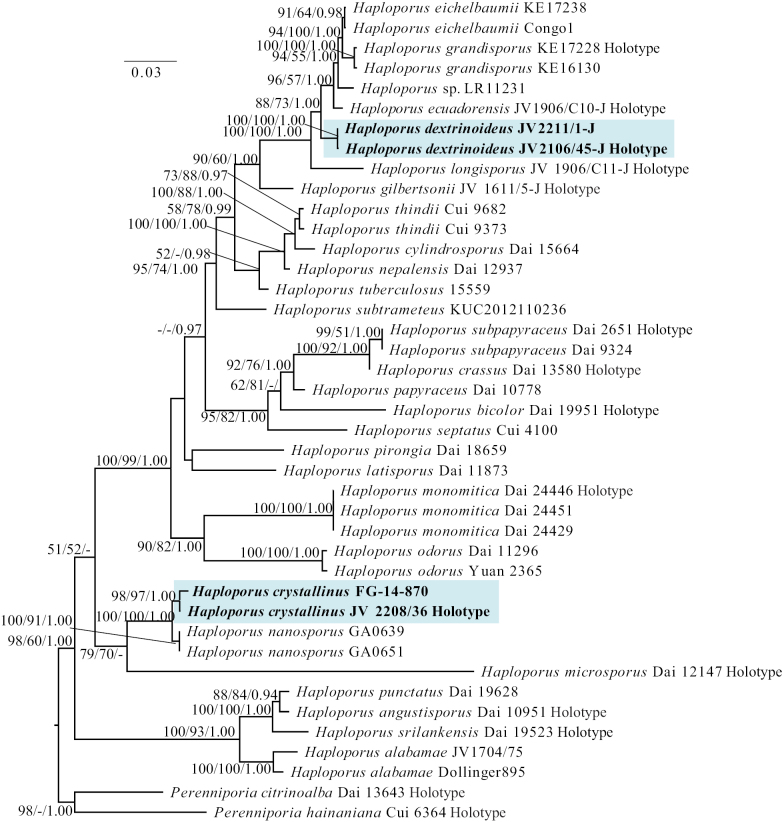
A Maximum Likelihood phylogenetic tree of *Haploporus* based on ITS, LSU and mtSSU gene fragments, with two specimens *Perenniporiacitrinoalba* and *P.hainaniana* used as outgroups. The new species *Haploporuscrystallinus* and *H.dextrinoideus* are shaded. Maximum Likelihood bootstrap values (≥ 50%)/Maximum Parsimony bootstrap values (≥ 50%)/Bayesian Posterior Probabilities (≥ 0.90) of each clade are indicated along branches. The scale bar left upper indicates the number of substitutions per site.

**Figure 2.**
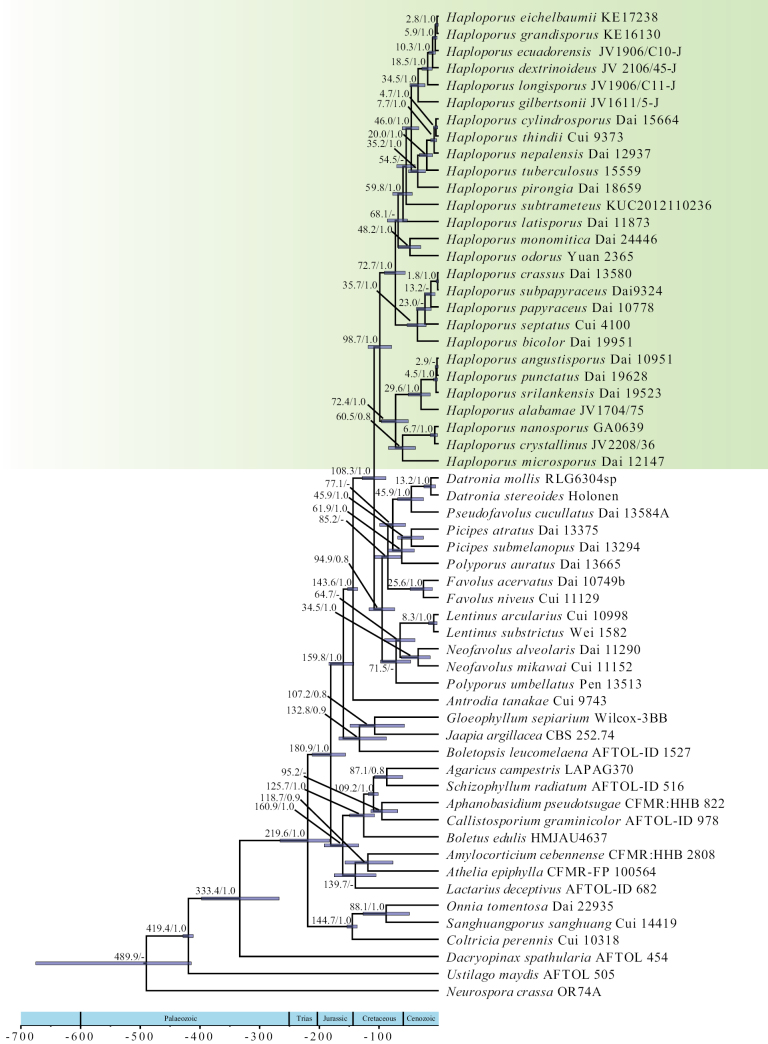
Estimated divergence of *Haploporus* generated by molecular clock analyses using the aligned dataset 2 of ITS and LSU sequences. Estimated mean divergence time (Mya) and Posterior Probabilities (PP) > 0.8 are annotated at the internodes. The 95% Highest Posterior Density (HPD) interval of divergence time estimates are marked by horizontal blue bars. The geological timescale is indicated at the bottom.

**Figure 3.**
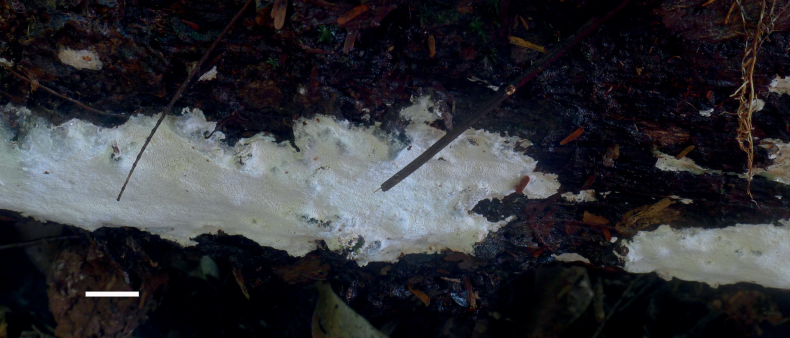
Basidiomata of *Haploporuscrystallinus* (Holotype, JV 2208/36). Scale bar: 1 cm.

The ITS, LSU and mtSSU partial sequences were aligned using MAFFT v.7 (Katoh and Standley 2013) and concatenated after excluding the poorly-aligned sites as dataset 1. The aligned dataset 1 was analysed using Maximum Likelihood (ML), Maximum Parsimony (MP) and Bayesian Inference (BI) phylogenetic analyses with RAxML v.8 (Stamatakis 2014), PAUP v.4.0b10 (Swofford 2002) and MrBayes v.3.2.7a (Ronquist et al. 2012), respectively, followed the previous studies (Zhao et al. 2021; Man et al. 2023). Additionally, the phylogenetic analyses of the aligned ITS, LSU and mtSSU partial sequences were also undertaken using RAxML v.8 (Suppl. materials 1: figs 1–3; Stamatakis 2014), respectively. The optimum model of the aligned dataset 1 was tested using the ModelTest-NG v.0.1.7 (Darriba et al. 2020). For ML and MP analyses, 1,000 bootstrap replications were carried out. For BI analysis, two million generations were conducted with random initial trees and the first 25% were set as burn-in.

### ﻿Divergence time analysis

Dataset 2, consisting of 58 ITS and LSU partial sequences, was used to infer the divergence times of the species in the genus *Haploporus* (Table 1, Suppl. material 1: table S1 and file S1). The divergence time was estimated with BEAST v.2.6.5 (Bouckaert et al. 2014), following previous studies (Liu et al. 2022; Zhao et al. 2022b). Four time points were selected for calibration and the offset age with a gamma distributed prior (scale = 20, shape = 1): 1) *Archaeomarasmiusleggettii*Hibbett et al. (1995, 1997) represented the divergence time of Agaricales at 90 Mya; 2) *Quatsinoporitescranhamii* S.Y. Sm. et al. (Smith et al. 2004; Berbee and Taylor 2010) represented the Hymenochaetaceae at 125 Mya; 3) *Paleopyrenomycitesdevonicus* Taylor et al. (Taylor et al. 1999, 2005) represented between Ascomycota and Basidiomycota at 400 Mya; and 4) the estimated mean crown age of Polyporales at 123.74 Mya (Ji et al. 2022). A total of 10,000,000 generations were set, first 20% being burn-in. The results of the log file and trees file were assessed using Tracer v.1.5 and TreeAnnotator v.2.6.5, respectively.

## ﻿Results

### ﻿Phylogeny

In this study, dataset 1, including a total of 41 sequences, was used to reconstruct the phylogenetic relationships of the genus *Haploporus* (Table 1, Fig. 1 and Suppl. material 1: file S1). The aligned dataset 1 has a length of 2,797 characters (ITS, 1–783 characters; LSU, 784–2063 characters; and mtSSU, 2064–2797 characters), of which 1,890 are constant characters, 266 are parsimony-uninformative characters and 641 are parsimony-informative characters. The Maximum Parsimony (MP) analysis yielded a tree of length 779, consistency index 0.4856, homoplasy index 0.5144, retention index 0.7658 and rescaled consistency index of 0.3719. The best model for the aligned dataset 1 was GTR + I + G in the Bayesian analysis and the average standard deviation of split frequencies was 0.004282. The phylograms of three analyses, ML, BI and MP, are similar in topology and the ML tree was selected to represent the phylogenetic relationships (Fig. 1).

The phylogenetic analyses indicated that *H.crystallinus* is clustered with *H.nanosporus* and *H.microsporus* and *H.dextrinoideus* formed an independent lineage in the *Haploporus* clade, closely related with *H.grandisporus*, *H.ecuadorensis*, *H.eichelbaumii* and *H.longisporus* (Fig. 1). BLAST results of ITS sequences with the top hit taxa were followed by phylogenetics analyses (Suppl. material 1: table S2).

### ﻿Divergence times of the *Haploporus*

In the present study, the molecular clock analysis suggested that the order Polyporales and genus *Haploporus* emerged at a mean stem of 159.8 Mya [95% higher posterior density (HPD) of 142.4–184.1 Mya] and 108.3 Mya (95% HPD of 88.5–128.2 Mya), respectively (Fig. 2). A total of 27 species of the genus *Haploporus* was estimated as molecular sequences in *H.brasiliensis* and *H.pileatus* were not available (Lira et al. 2018). The results suggested that species of the genus *Haploporus* may be diversified between 60.5 Mya and 1.8 Mya, during the Paleogene to Neogene in Cenozoic (Fig. 2 and Table 2). Amongst those species, 18 species occurred in the Neogene, accounting for 67% and only nine species originated in the Paleogene.

**Table 2.** Divergence times of estimated taxa in the genus *Haploporus*.

Species	Mean of stem in MCC tree (Mya)	95% HPD
*Haploporusalabamae*	29.6	14.1–51.2
*H.angustisporus*	2.9	1.1–5.9
*H.bicolor*	35.7	21.1–53.0
*H.crassus*	1.8	0.3–4.5
***H.crystallinus***	6.7	2.3–14.1
*H.cylindrosporus*	4.7	1.9–8.8
***H.dextrinoideus***	10.3	5.4–16.8
*H.ecuadorensis*	5.9	2.7–10.2
*H.eichelbaumii*	2.8	0.9–5.6
*H.gilbertsonii*	34.5	23.0–48.3
*H.grandisporus*	2.8	0.9–5.6
*H.latisporus*	59.8	52.7–86.0
*H.longisporus*	18.5	10.9–28.2
*H.microsporus*	60.5	39.1–84.1
*H.monomitica*	48.2	23.0–67.8
*H.nanosporus*	6.7	2.3–14.1
*H.nepalensis*	7.7	3.6–13.4
*H.odorus*	48.2	23.0–67.8
*H.papyraceus*	13.2	6.0–23.8
*H.pirongia*	35.2	22.1–50.5
*H.punctatus*	2.9	1.1–5.9
*H.septatus*	23.0	12.7–37.5
*H.srilankensis*	4.5	1.9–8.5
*H.subpapyraceus*	1.8	0.3–4.5
*H.subtrameteus*	54.5	44.4–77.1
*H.thindii*	4.7	1.9–8.8
*H.tuberculosus*	20.0	10.4–32.4

MCC represented as maximum clade credibility.

## ﻿Taxonomy

### ﻿ Haploporus crystallinus

Taxon classificationFungiPolyporalesPolyporaceae

H. Zhao, Vlasák & Yuan Yuan sp. nov.

689861A8-7E27-5827-9DEE-676126F6357C

MycoBank No: 849261

Figs 3 , 4

#### Etymology.

*crystallinus* (Lat.): Refers to the species having many crystals amongst the subiculum and tube trama.

#### Type.

French Guiana, Roura, Camp Cayman, rotten log on the road, 27 August 2022, JV 2208/36 (Holotype PRM, isotypes BJFC 039927 and JV 2208/36). GenBank: ITS = OQ919235, LSU = OQ919238, mtSSU = OQ919241.

Basidiomata resupinate, perennial, inseparable from the substrate, more or less corky when dry, up to 10 cm long, 3.5 cm wide and 4 mm thick at centre. Hymenophore pinkish-buff (5A3) to cream buff (4A4) when dry, with indistinct margin; pores angular to round, 5–7 per mm; dissepiments thick, entire. Subiculum darker than tubes, more or less corky. Tubes pinkish-buff (5A3), hard corky.

Hyphal system dimitic; generative hyphae with clamp connections, hyaline, thin-walled; skeletal hyphae thick-walled, frequently branched, distinctly dextrinoid in Melzer’s reagent, cyanophilous in Cotton Blue; tissues unchanged in 2% potassium hydroxide.

Subicular generative hyphae infrequent, hyaline, thin-walled, sometimes branched, 1.0–1.5 µm in diam.; skeletal hyphae dominant, with a narrow lumen to subsolid, usually branched, flexuous, interwoven, 0.8–2.0 µm in diam. Irregular-shaped and -sized crystals frequently present.

**Figure 4.**
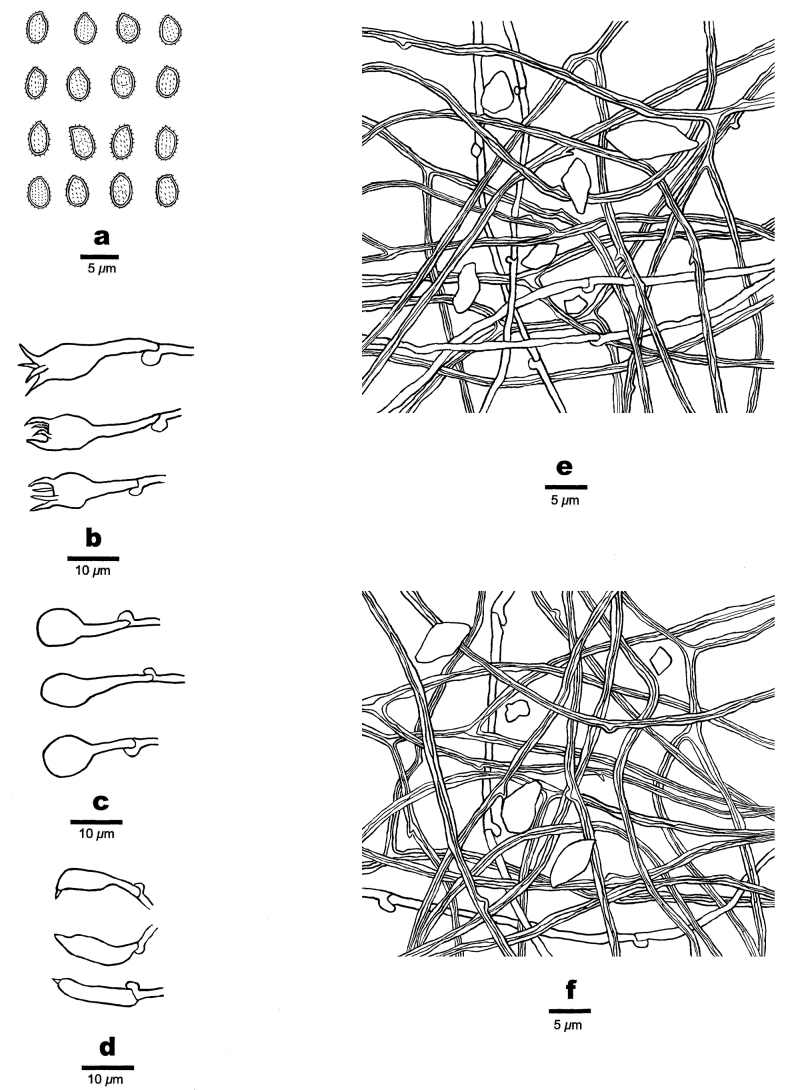
Microscopic characteristics of *Haploporuscrystallinus* (Holotype, JV 2208/36) **a** basidiospores **b** basidia **c** basidioles **d** cystidioles **e** hyphae from tube trama **f** hyphae from subiculum. Scale bars: 5 μm (**a**); 10 μm (**b–f**).

Tube tramal generative hyphae infrequent, hyaline, thin-walled, sometime branched, 1.0–1.5 µm in diam.; skeletal hyphae dominant, with a narrow lumen to subsolid, usually branched, flexuous, interwoven, 0.8–2.0 µm in diam. Cystidioles fusiform with a sharp tip, thin-walled, hyaline, 12.0–21.0 × 3.0–4.5 µm. Basidia more or less capitate to pyriform, with four sterigmata and a clamp connection at base, 17.5–27.0 × 6.5–9.0 µm; basidioles capitate to pyriform, almost the same size of basidia. Dendrohyphidia absent. Irregular-shaped and -sized crystals frequently present.

Basidiospores ellipsoid, slightly thick-walled, tuberculate, hyaline, some with a guttule, dextrinoid in Melzer’s reagent, cyanophilous in Cotton Blue, (3.8–)4.0–5.5 × (2.1–)2.6–3.5(–3.8) µm, L (arithmetic average length) = 4.60 µm, W (arithmetic average width) = 3.03 µm, Q (L/W ratio) = 1.52 (n = 30/1).

#### Distribution and ecology.

*Haploporuscrystallinus* is distributed in French Guiana and growing on rotten unidentified angiosperm log; causes a white rot.

### ﻿ Haploporus dextrinoideus

Taxon classificationFungiPolyporalesPolyporaceae

H. Zhao, Vlasák & Yuan Yuan sp. nov.

6736A1D9-4DE1-5262-BCDF-515CCCB11171

MycoBank No: 849262

Figs 5 , 6

#### Etymology.

*dextrinoideus* (Lat.): Refers to the species having dextrinoid hyphae.

#### Type.

Ecuador, Papallacta Termas, 3,300 m standing dead tree, 15 June 2021, Josef Vlasák Jr., JV 2106/45-J (Holotype PRM, isotypes BJFC 038566 and JV). GenBank: ITS = OQ919236, LSU = OQ919239.

Basidiomata resupinate, annual, inseparable from the substrate, more or less corky when dry, up to 5.0 cm long, 3.0 cm wide and 0.4 mm thick at centre. Hymenophore cream bubalinus (4A2/3) to pinkish-buff (5A3) when dry, margin indistinct; pores angular to round, 1–3 per mm; dissepiments thick, entire. Subiculum slightly darker than tubes, more or less corky, up to 0.2 mm thick. Tubes pinkish-buff (5A3), hard corky.

**Figure 5.**
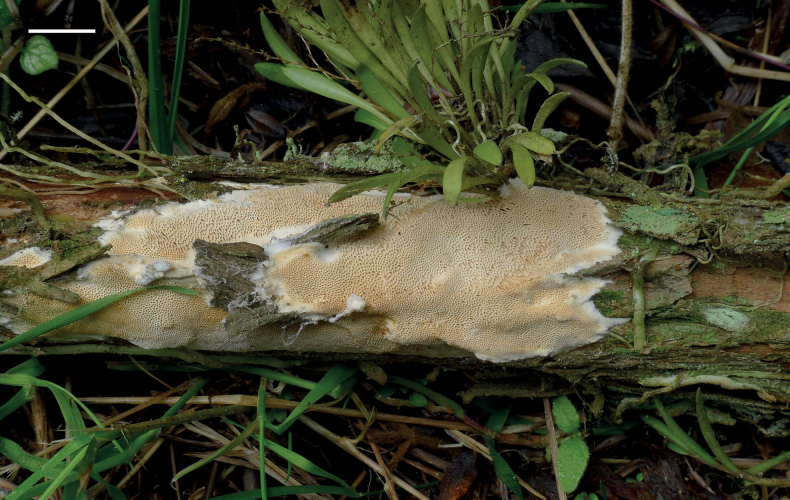
Basidiomata of *Haploporusdextrinoideus* (Holotype, JV 2206/45-J). Scale bar: 1 cm.

Hyphal system dimitic; generative hyphae with clamp connections; skeletal hyphae thick-walled, branched, dextrinoid in Melzer’s reagent, cyanophilous in Cotton Blue; tissues unchanged in 2% potassium hydroxide.

Subicular generative hyphae hyaline, thin-walled, sometimes branched, 1.0–3.0 µm in diam.; skeletal hyphae dominant, with a wide lumen, usually branched, flexuous, interwoven, 1.5–3.0 µm in diam.

Tube tramal generative hyphae hyaline, thin-walled, usually branched, 1.0–2.5 µm in diam.; skeletal hyphae dominant, with a wide lumen, usually branched, flexuous, distinctly interwoven, 1.0–3.0 µm in diam. Cystidioles fusiform with a sharp tip, thin-walled, hyaline, 19.0–35.0 × 4.5–6.5 µm. Basidia more or less capitate to pyriform, with four sterigmata and a clamp connection at base, sometimes with a few small guttules, 21.0–34.0 × 7.0–14.0 µm; basidioles capitate to pyriform, almost the same size of basidia. Dissepimental hyphae thick-walled with one or four simple septa. Dendrohyphidia present amongst hymenium, thin-walled, hyaline. Large and irregularly-shaped crystals sometimes present amongst trama.

**Figure 6.**
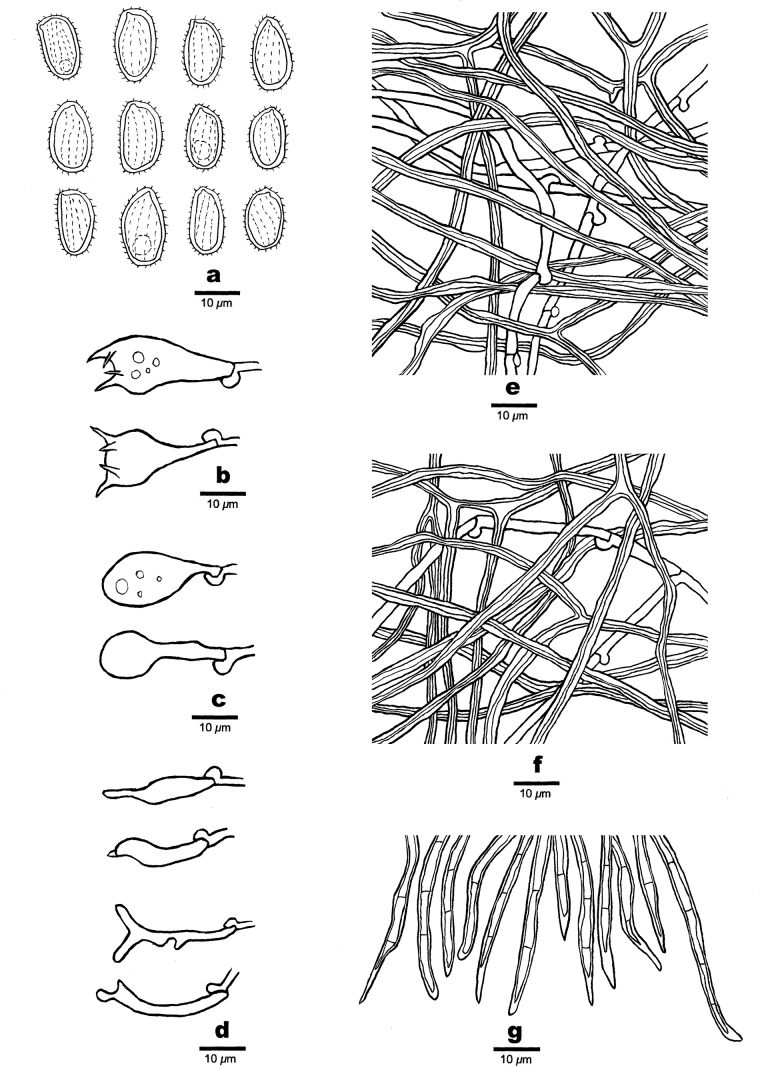
Microscopic characteristics of *Haploporusdextrinoideus* (Paratype, JV 2211/1-J) **a** basidiospores **b** basidia **c** basidioles and cystidioles **d** dendrohyphidia **e** hyphae from tube trama **f** hyphae from subiculum **g** dissepiment hyphae. Scale bars: 10 μm (**a–g**).

Basidiospores oblong to ellipsoid, thick-walled, tuberculate, hyaline, some with a guttule, dextrinoid in Melzer’s reagent, cyanophilous in Cotton Blue, (12.0–)13.2–19.0 × (5.0–)6.0–9.0 µm, L = 15.43 µm, W = 7.78 µm, Q = 1.98–2.16 (n = 60/2).

#### Additional material studied.

Ecuador, Papallacta Termas, 3,300 m, on unidentified angiosperm, November 2022, Josef Vlasák Jr., JV 2211/1-J.

#### Distribution and ecology.

*Haploporusdextrinoideus* is known from Ecuador high mountains, 3,300 m and growing on dead unidentified angiosperm trees; causes a white rot.

## ﻿Discussion

Phylogenetic analyses, based on a combined dataset 1 (ITS + LSU + mtSSU sequences), indicates that *H.crystallinus* forms a sister group to *H.nanosporus* and *H.microsporus* with strong support. *H.nanosporus* differs from *H.crystallinus* by rarely branched skeletal hyphae, wider generative hyphae (1.5–2.0 µm vs. 1.0–1.5 µm) and indextrinoid basidiospores (David and Rajchenberg 1992). Additionally, *H.nanosporus* is distributed in Africa (David and Rajchenberg 1992; Decock et al. 2021), while *H.crystallinus* is found in French Guiana, South America. *Haploporusmicrosporus* differs from *H.crystallinus* by smaller pores (7–9 vs. 5–7 per mm) and annual basidiomata (Zhou et al. 2019). Specimen *Haploporus* sp. FG-14-870 (GenBank numbers: MT782653 and MT777443), collected by Cony Decock on 7 April 2014 in French Guiana, shows ITS sequence similarity with *H.crystallinus*JV 2208/36 over 99%, forming together a clade in the phylogenetic tree. Therefore, *Haploporus* sp. FG-14-870 is another specimen of *Haploporuscrystallinus*.

Phylogenetic analyses also suggest that *H.dextrinoideus* forms a clade with the *H.eichelbaumii*, *H.grandisporus*, *H.ecuadorensis* and *H.longisporus* (Fig. 1). *Haploporuseichelbaumii* differs from *H.dextrinoideus* by smaller and indextrinoid basidiospores [11.0–14.0 × 5.3–6.5 µm vs. 13.2–19.0 × 6.0–9.0 µm; Decock et al. (2021)]. *Haploporusgrandisporus* differs from *H.dextrinoideus* by narrower skeletal hyphae (1.3–1.8 µm vs. 1.5–3.0 µm), smaller basidia and basidioles (18.0–20.0 × 8.0–13.0 µm vs. 21.0–34.0 × 7.0–14.0 µm) and indextrinoid basidiospores (Decock et al. 2021). *Haploporusecuadorensis* differs from *H.dextrinoideus* by wider basidioles and basidia (40.0–45.0 µm vs. 19.0–35.0 µm) and hyphae and basidiospores neither amyloid nor dextrinoid (Man et al. 2023). *Haploporuslongisporus* differs from *H.dextrinoideus* by the indextrinoid skeletal hyphae and basidiospores (Zhou et al. 2021).

In addition, two species, *H.brasiliensis* and *H.pileatus*, were described in Brazil from South America without molecular data (Lira et al. 2018). *Haploporusbrasiliensis* differs from *H.crystallinus* by annual basidiomata, larger pores (1–3 per mm vs. 5–7 per mm), thin dissepiments, wider subicular generative hyphae (2.0–2.5 µm vs. 1.0–1.5 µm) and larger, long-ellipsoid basidiospores [6.0–8.0 × 4.0–5.0 µm vs. 4.0–5.5 × 2.6–3.5 µm; Lira et al. (2018)]. *Haploporuspileatus* differs from *H.crystallinus* by pileate basidiomata, larger pores (3–4 per mm vs. 5–7 per mm), thin dissepiments, cylindrical and larger basidiospores [9.0–10.0 × 4.0–5.0 µm vs. 4.0–5.5 × 2.6–3.5 µm; Lira et al. (2018)]. *Haploporusbrasiliensis* differs from *H.dextrinoideus* by smaller basidiospores (6.0–8.0 × 4.0–5.0 µm vs. 13.2–19.0 × 6.0–9.0 µm) and basidia [12.0–15.0 × 4.0–6.0 µm vs. 21.0–34.0 × 7.0–14.0 µm; Lira et al. (2018)]. *Haploporuspileatus* differs from *H.dextrinoideus* by smaller pores (3–4 per mm vs. 1–3 per mm), smaller basidiospores (9.0–10.0 × 4.0–5.0 µm vs. 13.2–19.0 × 6.0–9.0 µm) and basidia [14.0–24.0 × 5.0–7.0 µm vs. 21.0–34.0 × 7.0–14.0 µm; Lira et al. (2018)].

He et al. (2019) estimated the order Polyporales originated at a mean stem of 138 Mya and Ji et al. (2022) suggested that Polyporales originated in the Early Cretaceous, with a mean stem of 141.81 Mya (95% HPD of 102.35–191.91 Mya). Our molecular clock analysis showed that Polyporales originated in a mean stem of 159.8 Mya (95% HPD of 142.4–184.1 Mya), which overlapped previous studies (He et al. 2019; Ji et al. 2022). The genus *Haploporus* emerged at a mean stem of 108.3 Mya (95% HPD of 88.5–128.2 Mya), earlier than the genus *Polyporus* and its allied genera (Zhou et al. 2016; Ji et al. 2022). Species of the genus *Haploporus* probably diversified between 60.5 Mya and 1.8 Mya and 18 species occurred in the Neogene, indicating that species were rapidly diversified in this period. A similar result is evident in the genus *Onnia*, where most species also appeared in the Neogene (Zhao et al. 2022b).

The study reconstructed the phylogenetic relationships of the genus *Haploporus*, described two new species based on the molecular fragments and morphological evidence. Molecular clock analysis provided insight into the divergence times of *Haploporus* species.

### ﻿Key to species of *Haploporus*

**Table:** 

1	Hyphal system monomitic	***H.monomitica***
1a	Hyphal system dimitic to trimitic	**2**
2	Basidiospores < 8 µm long	**3**
2a	Basidiospores > 8 µm long	**7**
3	Pores 1–3 per mm	***H.brasiliensis***
3a	Pores > 3 per mm	**4**
4	Cystidioles absent	**5**
4a	Cystidioles present	**6**
5	Basidiomata annual to perennial, resupinate; pore 7–8 per mm; skeletal hyphae dextrinoid; basidiospores ellipsoid	***H.nanosporus***
5a	Basidiomata perennial, effused-reflexed to pileate; pore 3–5 per mm; skeletal hyphae IKI-; basidiospores ovoid	***H.odorus***
6	Basidiomata perennial; pore 5–7 per mm	***H.crystallinus***
6a	Basidiomata annual; pore 7–9 per mm	***H.microsporus***
7	Skeletal hyphae dextrinoid	**8**
7a	Skeletal hyphae non-dextrinoid	**9**
8	Basidiomata annual; pore 1–3 per mm; basidiospores 13.2–19.0 × 6.0–9.0 µm	***H.dextrinoideus***
8a	Basidiomata perennial; pore 4–5 per mm; basidiospores 8.5–11 × 4–5.2 μm	***H.srilankensis***
9	Basidiospores cylindrical	***H.thindii***
9a	Basidiospores oblong ellipsoid to ellipsoid	***H.subtrameteus***
10	Hyphal system trimitic	**11**
10a	Hyphal system dimitic	**13**
11	Skeletal hyphae dextrinoid	***H.tuberculosus***
11a	Skeletal hyphae non-dextrinoid	**12**
12	Basidiospores ovoid to ellipsoid	***H.alabamae***
12a	Basidiospores oblong-ellipsoid to cylindrical	***H.pirongia***
13	Cystidioles absent	**14**
13a	Cystidioles present	**16**
14	Basidiomata pileate	***H.pileatus***
14a	Basidiomata resupinate	**15**
15	Pores 4–5 per mm, basidiospores cylindrical, 10–11.5 × 4.5–5 µm	***H.cylindrosporus***
15a	Pores 1.5–4 per mm, basidiospores ellipsoid to oblong, 10–15 × 5–6.8 µm	***H.eichelbaumii***
16	Dendrohyphidia present	**17**
16a	Dendrohyphidia absent	**21**
17	Pores 5–7 per mm	***H.bicolor***
17a	Pores < 4 per mm	**18**
18	Basidiospores cylindrical	**19**
18a	Basidiospores ellipsoid to oblong	**20**
19	Basidiospores 18.2–22 × 7–9 µm	***H.longisporus***
19a	Basidiospores 13–15 × 5–6 µm	***H.papyraceus***
20	Hyphal system trimitic, skeletal hyphae dextrinoid	***H.grandisporus***
20a	Hyphal system dimitic, skeletal hyphae non-dextrinoid	***H.ecuadorensis***
21	Pores > 3 per mm	**22**
21a	Pores < 3 per mm	**26**
22	Pores 5–6 per mm	***H.septatus***
22a	Pores 3–5 per mm	**23**
23	Skeletal hyphae non-dextrinoid	***H.crassus***
23a	Skeletal hyphae dextrinoid	**24**
24	Cystidioles without septum	***H.angustisporus***
24a	Cystidioles with a simple septum	**25**
25	Basidiospores 9–10.8 × 3.8–5 µm	***H.punctatus***
25a	Basidiospores 9–12 × 5.5–8 µm	***H.subpapyraceus***
26	Basidiospores 9–10 µm wide	***H.latisporus***
26a	Basidiospores < 9 µm wide	**27**
27	Basidiospores 12–15 × 6–8 µm	***H.gilbertsonii***
27a	Basidiospores 8.5–11.5 × 4.5–6.5 µm	***H.nepalensis***

## Supplementary Material

XML Treatment for Haploporus crystallinus

XML Treatment for Haploporus dextrinoideus

## ﻿Additional information

### Author ORCIDs

Heng Zhao https://orcid.org/0000-0003-2938-5613

Josef Vlasák https://orcid.org/0000-0002-5363-4459

Yuan Yuan https://orcid.org/0000-0001-6674-9848

### Data availability

The sequences are deposited in the GenBank database (Table 1).

## Supplementary materials

Supplementary material 1 The supplementary materilas in this study Data type (docx.file, fasta.file) Explanation note **figure S1.** A Maximum Likelihood phylogenetic tree of *Haploporus*, based on ITS sequences, with two specimens *Perenniporiacitrinoalba* and *P.hainaniana* used as outgroups. The new species *Haploporuscrystallinus* and *H.dextrinoideus* are in bold. Maximum Likelihood bootstrap values (≥ 50%) of each clade is indicated along branches. The scale bar left upper indicates the number of substitutions per site. **figure S2.** A Maximum Likelihood phylogenetic tree of *Haploporus*, based on LSU sequences, with two specimens *Perenniporiacitrinoalba* and *P.hainaniana* used as outgroups. The new species *Haploporuscrystallinus* and *H.dextrinoideus* are in bold. Maximum Likelihood bootstrap values (≥ 50%) of each clade is indicated along branches. The scale bar left upper indicates the number of substitutions per site. **figure S3.** A Maximum Likelihood phylogenetic tree of *Haploporus*, based on mtSSU sequences, with two specimens *Perenniporiacitrinoalba* and *P.hainaniana* used as outgroups. The new species *Haploporuscrystallinus* and *H.dextrinoideus* are in bold. Maximum Likelihood bootstrap values (≥ 50%) of each clade is indicated along branches. The scale bar left upper indicates the number of substitutions per site. **table S1.** Specimens and GenBank accession numbers used in this study. **table S2.** The top hits of the new species, based on BLAST of ITS sequences from GenBank. **file S1.** All the aligned sequences. https://binary.pensoft.net/file/883143This dataset is made available under the Open Database License (http://opendatacommons.org/licenses/odbl/1.0/). The Open Database License (ODbL) is a license agreement intended to allow users to freely share, modify, and use this Dataset while maintaining this same freedom for others, provided that the original source and author(s) are credited.Heng Zhao, Josef Vlasák, Yuan Yuan

## References

[B1] Anonymous (1969) Flora of British fungi. Colour identification chart. Her Majesty’s Stationery Office, London.

[B2] Berbee ML Taylor JW (2010) Dating the molecular clock in fungi – how close are we? Fungal Biology Reviews 24(1–2): 1–16. 10.1016/j.fbr.2010.03.001

[B3] Bouckaert R Heled J Kühnert D Vaughan T Wu CH Xie D Suchard MA Rambaut A Drummond AJ (2014) BEAST 2: A software platform for Bayesian evolutionary analysis. PLoS Computational Biology 10(4): e1003537. 10.1371/journal.pcbi.1003537 PMC3985171 24722319

[B4] Cao Y Wu SH Dai YC (2012) Species clarification of the prize medicinal *Ganoderma* mushroom “Lingzhi”. Fungal Diversity 56(1): 49–62. 10.1007/s13225-012-0178-5

[B5] Chen JJ Cui BK Zhou LW Korhonen K Dai YC (2015) Phylogeny, divergence time estimation, and biogeography of the genus *Heterobasidion* (Basidiomycota, Russulales). Fungal Diversity 71(1): 185–200. 10.1007/s13225-014-0317-2

[B6] Chen JJ Cui BK He SH Cooper JA Barrett MD Chen JL Dai YC (2016) Molecular phylogeny and global diversity of the remarkable genus *Bondarzewia* (Basidiomycota, Russulales). Mycologia 108(4): 697–708. 10.3852/14-216 27091389

[B7] Cui YY Cai Q Tang LP Liu JW Yang ZL (2018) The family Amanitaceae: Molecular phylogeny, higher-rank taxonomy and the species in China. Fungal Diversity 91(1): 5–230. 10.1007/s13225-018-0405-9

[B8] Cui BK Li HJ Ji X Zhou JL Song J Si J Yang ZL Dai YC (2019) Species diversity, taxonomy and phylogeny of Polyporaceae (Basidiomycota) in China. Fungal Diversity 97(1): 137–392. 10.1007/s13225-019-00427-4

[B9] Dai YC (2010) Hymenochaetaceae (Basidiomycota) in China. Fungal Diversity 45(1): 131–343. 10.1007/s13225-010-0066-9

[B10] Dai YC (2012a) Polypore diversity in China with an annotated checklist of Chinese polypores. Mycoscience 53(1): 49–80. 10.1007/s10267-011-0134-3

[B11] Dai YC (2012b) Pathogenic wood-decaying fungi on woody plants in China. Mycosystema 31: 493–509.

[B12] Dai YC Niemelä T Kinnunen J (2002) The polypore genera *Abundisporus* and *Perenniporia* (Basidiomycota) in China, with notes on *Haploporus*. Annales Botanici Fennici 39: 169–182.

[B13] Dai YC Wei YL Wang Z (2004) Wood-inhabiting fungi in southern China 2. Polypores from Sichuan Province. Annales Botanici Fennici. Finnish Zoological and Botanical Publishing Board, 319–329.

[B14] Dai YC Cui BK Yuan HS Li BD (2007) Pathogenic wood-decaying fungi in China. Forest Pathology 37(2): 105–120. 10.1111/j.1439-0329.2007.00485.x

[B15] Dai YC Yang ZL Cui BK Wu G Yuan HS Zhou LW He SH Ge ZW Wu F Wei YL Yuan Y Si J (2021) Diversity and systematics of the important macrofungi in Chinese forests. Mycosystema 40: 770–805.

[B16] Darriba D Posada D Kozlov AM Stamatakis A Morel B Flouri T (2020) ModelTest-NG: A new and scalable tool for the selection of DNA and protein evolutionary models. Molecular Biology and Evolution 37(1): 291–294. 10.1093/molbev/msz189 31432070 PMC6984357

[B17] David A Rajchenberg M (1992) West African polypores. New species and combinations. Mycotaxon 45: 131–148.

[B18] Decock CA Wagara I Balezi A Yombiyeni P (2021) *Haploporus* (Basidiomycota, Polyporales) in sub-Saharan Africa: Poriaeichelbaumii, a long-forgotten name, is reinstated in *Haploporus* and *H.grandisporus* sp. nov. is proposed. Mycological Progress 20(2): 149–168. 10.1007/s11557-020-01660-x

[B19] Hawksworth DL Lücking R (2017) Fungal diversity revisited: 2.2 to 3.8 million species. Microbiology Spectrum 5(4): 1–4. 10.1128/microbiolspec.FUNK-0052-2016 PMC11687528 28752818

[B20] He MQ Zhao RL Hyde KD Begerow D Kemler M Yurkov A McKenzie EHC Raspé O Kakishima M Sánchez-Ramírez S Vellinga EC Halling R Papp V Zmitrovich IV Buyck B Ertz D Wijayawardene NN Cui BK Schoutteten N Liu XZ Li TH Yao YJ Zhu XY Liu AQ Li GJ Zhang MZ Ling ZL Cao B Antonín V Boekhout T da Silv BDB de Crop E Decock C Dima B Dutta AK Fell JW Geml J Ghobad-Nejhad M Giachini AJ Gibertoni TB Gorjón SP Haelewaters D He SH Hodkinson BP Horak E Hoshino T Justo A Lim YW Menolli N Mešić A Moncalvo JM Mueller GM Nagy LG Nilsson RH Noordeloos M Nuytinck J Orihara T Ratchadawan C Rajchenberg M Silva-Filho AGS Sulzbacher MA Tkalčec Z Valenzuela R Verbeken A Vizzini A Wartchow F Wei TZ Weiß M Zhao CL Kirk PM (2019) Notes, outline and divergence times of Basidiomycota. Fungal Diversity 99(1): 105–367. 10.1007/s13225-019-00435-4

[B21] Hibbett DS Grimaldi D Donoghue MJ (1995) Cretaceous mushrooms in amber. Nature 377(6549): 487–487. 10.1038/377487a0

[B22] Hibbett DS Grimaldi D Donoghue MJ (1997) Fossil mushrooms from Miocene and Cretaceous ambers and the evolution of Homobasidiomycetes. American Journal of Botany 84(7): 981–991. 10.2307/2446289 21708653

[B23] Ji X Zhou JL Song CG Xu TM Wu DM Cui BK (2022) Taxonomy, phylogeny and divergence times of *Polyporus* (Basidiomycota) and related genera. Mycosphere: Journal of Fungal Biology 13(1): 1–52. 10.5943/mycosphere/13/1/1

[B24] Katoh K Standley DM (2013) MAFFT multiple sequence alignment software version 7: Improvements in performance and usability. Molecular Biology and Evolution 30(4): 772–780. 10.1093/molbev/mst010 23329690 PMC3603318

[B25] Kirk PM Cannon PF Minter DW Stalpers JA (2008) Ainsworth & Bisby’s Dictionary of the Fungi (10^th^ edn.). CAB International, 771 pp. 10.1017/S0269915X03001204

[B26] Li J Dai YC Yuan HS (2007) A new species of *Haploporus* (Basidiomycotina) from China. Mycotaxon 99: 181–187.

[B27] Lira C Nogueira-Melo G Ryvarden L Gibertoni T (2018) Two new species of *Haploporus* from Brazil. Synopsis Fungorum 38: 62–65.

[B28] Liu ZB Wu YD Zhao H Lian YP Wang YR Wang CG Mao WL Yuan Y (2022) Outline, divergence Times, and phylogenetic analyses of Trechisporales (Agaricomycetes, Basidiomycota). Frontiers in Microbiology 13: e818358. 10.3389/fmicb.2022.818358 PMC9083364 35547118

[B29] Man XW Dai YC Bian LS Zhou M Zhao H Vlasák J (2023) Two new species of *Haploporus* (Polyporales, Basidiomycota) from China and Ecuador based on morphology and phylogeny. Frontiers in Cellular and Infection Microbiology 13: e1133839. 10.3389/fcimb.2023.1133839 PMC9990840 36896189

[B30] Petersen JH (1996) The Danish Mycological Society’s colour-chart. Foreningen til Svampekundskabens Fremme, Greve, 6 pp.

[B31] Piątek M (2003) *Haploporustuberculosus*, a new polypore genus and species in Belarus, with a new combination in *Haploporus*. Polish Botanical Journal 48: 81–83.

[B32] Piątek M (2005) Taxonomic position and world distribution of *Pachykytosporananospora* (Polyporaceae). Annales Botanici Fennici 42: 23–25.

[B33] Ronquist F Teslenko M Van Der Mark P Ayres DL Darling A Höhna S Larget B Liu L Suchard MA Huelsenbeck JP (2012) MrBayes 3.2: Efficient Bayesian phylogenetic inference and model choice across a large model space. Systematic Biology 61(3): 539–542. 10.1093/sysbio/sys029 22357727 PMC3329765

[B34] Ryvarden L Gilbertson RL (1993) European polypores. Part 1. Synopsis Fungorum 6: 1–387.

[B35] Shen LL Chen JJ Wang M Cui BK (2016) Taxonomy and multi-gene phylogeny of *Haploporus* (Polyporales, Basidiomycota). Mycological Progress 15(7): 731–742. 10.1007/s11557-016-1203-y

[B36] Singer R (1944) Notes on taxonomy and nomenclature of the polypores. Mycologia 36(1): 65–69. 10.1080/00275514.1944.12017529

[B37] Smith SY Currah RS Stockey RA (2004) Cretaceous and Eocene poroid hymenophores from Vancouver Island, British Columbia. Mycologia 96(1): 180–186. 10.1080/15572536.2005.11833010 21148842

[B38] Stamatakis A (2014) RAxML version 8: A tool for phylogenetic analysis and post-analysis of large phylogenies. Bioinformatics 30(9): 1312–1313. 10.1093/bioinformatics/btu033 24451623 PMC3998144

[B39] Swofford DL (2002) PAUP*: Phylogenetic Analysis Using Parsimony (* and Other Methods); Version 4.0b10. Sinauer Associates, Sunderland.

[B40] Taylor TN Hass H Kerp H (1999) The oldest fossil ascomycetes. Nature 399(6737): 648–648. 10.1038/21349 10385115

[B41] Taylor TN Hass H Kerp H Krings M Hanlin RT (2005) Perithecial ascomycetes from the 400 million year old Rhynie chert: An example of ancestral polymorphism. Mycologia 97(1): 269–285. 10.1080/15572536.2006.11832862 16389979

[B42] Varga T Krizsán K Földi C Dima B Sánchez-García M Sánchez-Ramírez S Szöllősi GJ Szarkándi JG Papp V Albert L Andreopoulos W Angelini C Antonín V Barry KW Bougher NL Buchanan P Buyck B Bense V Catcheside P Chovatia M Cooper J Dämon W Desjardin D Finy P Geml J Haridas S Hughes K Justo A Karasiński D Kautmanova I Kiss B Kocsubé S Kotiranta H LaButti KM Lechner BE Liimatainen K Lipzen A Lukács Z Mihaltcheva S Morgado LN Niskanen T Noordeloos ME Ohm RA Ortiz-Santana B Ovrebo C Rácz N Riley R Savchenko A Shiryaev A Soop K Spirin V Szebenyi C Tomšovský M Tulloss RE Uehling J Grigoriev IV Vágvölgyi C Papp T Martin FM Miettinen O Hibbett DS Nagy LG (2019) Megaphylogeny resolves global patterns of mushroom evolution. Nature Ecology & Evolution 3(4): 668–678. 10.1038/s41559-019-0834-1 30886374 PMC6443077

[B43] Wang K Krik PM Yao YJ (2019) The development trends in taxonomy, with a special reference to fungi. Journal of Systematics and Evolution 58(4): 406–412. 10.1111/jse.12538

[B44] Wu G Feng B Xu J Zhu XT Li YC Zeng NK Hosen MI Yang ZL (2014) Molecular phylogenetic analyses redefine seven major clades and reveal 22 new generic clades in the fungal family Boletaceae. Fungal Diversity 69(1): 93–115. 10.1007/s13225-014-0283-8

[B45] Wu F Zhou LW Vlasák J Dai YC (2022a) Global diversity and systematics of Hymenochaetaceae with poroid hymenophore. Fungal Diversity 113(1): 1–192. 10.1007/s13225-021-00496-4

[B46] Wu F Man XW Tohtirjap A Dai YC (2022b) A comparison of polypore fungal and species composition in forest ecosystems of China, North America, and Europe. Forest Ecosystems 9: e100051. 10.1016/j.fecs.2022.100051

[B47] Yuan Y Ji XH Chen JJ Dai YC (2017) Three new species of *Megasporia* (Polyporales, Basidiomycota) from China. MycoKeys 20: 37–50. 10.3897/mycokeys.20.11816

[B48] Zhao CL Cui BK (2013) Morphological and molecular identification of four new resupinate species of *Perenniporia* (Polyporales) from southern China. Mycologia 105(4): 945–958. 10.3852/12-201 23449076

[B49] Zhao H Zhu J Zong TK Liu XL Ren LY Lin Q Qiao M Nie Y Zhang ZD Liu XY (2021) Two new species in the family Cunninghamellaceae from China. Mycobiology 49(2): 142–150. 10.1080/12298093.2021.1904555 PMC10635138 37970189

[B50] Zhao H Dai YC Liu XY (2022a) Outline and divergence time of subkingdom Mucoromyceta: two new phyla, five new orders, six new families and seventy-three new species. bioRxiv. 10.1101/2022.07.05.498902

[B51] Zhao H Zhou M Liu XY Wu F Dai YC (2022b) Phylogeny, divergence time estimation and biogeography of the genus *Onnia* (Basidiomycota, Hymenochaetaceae). Frontiers in Microbiology 13: e907961. 10.3389/fmicb.2022.907961 PMC9301299 35875515

[B52] Zhou JL Zhu L Chen H Cui BK (2016) Taxonomy and phylogeny of *Polyporus* group *Melanopus* (Polyporales, Basidiomycota) from China. PLoS ONE 11: e0159495. 10.1371/journal.pone.0159495 PMC4972403 27486931

[B53] Zhou M Wang L May TW Vlasák J Chen JJ Dai YC (2019) Phylogeny and diversity of *Haploporus* (Polyporaceae, Basidiomycota). MycoKeys 54: 77–98. 10.3897/mycokeys.54.34362 31244548 PMC6584150

[B54] Zhou M Dai YC Vlasák J Yuan Y (2021) Molecular phylogeny and global diversity of the genus *Haploporus* (Polyporales, Basidiomycota). Journal of Fungi 7(2): 96. 10.3390/jof7020096 33572799 PMC7912676

