# Supplementary material for: ﻿Outline, phylogenetic and divergence times analyses of the genus Haploporus (Polyporales, Basidiomycota): two new species are proposed
Source: MycoKeys. 2023 Jul 24;98:233–52. doi: 10.3897/mycokeys.98.105684 (PMC10390986; doi:10.3897/mycokeys.98.105684)
Supplement: Supplementary material 1 — The supplementary materilas in this study [file mycokeys-98-233-s001.zip › Supplementary Materials/Figure S1-3.docx]

**Figure S1.** A Maximum Likelihood phylogenetic tree of *Haploporus* based on ITS sequences, with two specimens of *Perenniporia* *citrinoalba* and *P. hainaniana* used as outgroups. The new species *Haploporus* *crystallinus* and *H. dextrinoideus* are bolded. Maximum Likelihood bootstrap values (≥50 %) of each clade is indicated along branches. A scale bar left upper indicates the number of substitutions per site.

**Figure S2.** A Maximum Likelihood phylogenetic tree of *Haploporus* based on LSU sequences, with two specimens of *Perenniporia citrinoalba* and *P. hainaniana* used as outgroups. The new species *Haploporus* *crystallinus* and *H. dextrinoideus* are bolded. Maximum Likelihood bootstrap values (≥50 %) of each clade is indicated along branches. A scale bar left upper indicates the number of substitutions per site.

**Figure S3.** A Maximum Likelihood phylogenetic tree of *Haploporus* based on mtSSU sequences, with two specimens of *Perenniporia citrinoalba* and *P. hainaniana* used as outgroups. The new species *Haploporus* *crystallinus* and *H. dextrinoideus* are bolded. Maximum Likelihood bootstrap values (≥50 %) of each clade is indicated along branches. A scale bar left upper indicates the number of substitutions per site.
