# Supplementary material for: ﻿Outline, phylogenetic and divergence times analyses of the genus Haploporus (Polyporales, Basidiomycota): two new species are proposed
Source: MycoKeys. 2023 Jul 24;98:233–52. doi: 10.3897/mycokeys.98.105684 (PMC10390986; doi:10.3897/mycokeys.98.105684)
Supplement: Supplementary material 1 — The supplementary materilas in this study [file mycokeys-98-233-s001.zip › Supplementary Materials/Table S2.docx]

**Table S2.** The top hits of the new species based on BLAST of ITS sequences from GenBank.

| Query | Description | Query cover (%) | Percent identity (%) |
| --- | --- | --- | --- |
| *Haploporus crystallinus* | *Haploporus* sp. FG-14-870 | 98 | 99.38 |
|  | *Haploporus* sp. FG-14-820 | 99 | 97.55 |
|  | *H. nanosporus* GA-09-514 | 98 | 96.77 |
|  | *H. nanosporus* GA-06-39 | 98 | 96.77 |
|  | *H. nanosporus* GA-06-51 | 98 | 96.77 |
|  | *H. nanosporus* GA-09-521 | 98 | 96.77 |
|  | *Haploporus* sp. FG-13-659 | 93 | 89.73 |
|  | *H. microsporus* Dai 12417 | 100 | 84.03 |
| *H. dextrinoideus* | *H. eichelbaumii* KE-18-295 | 100 | 90.23 |
|  | *H. eichelbaumii* KE-17-238 | 100 | 90.23 |
|  | *H. eichelbaumii* KE-17-240 | 100 | 90.23 |
|  | *H. eichelbaumii* MA-LR-11411 | 100 | 90.23 |
|  | *H. ecuadorensis* JV 1906/C10-J | 100 | 89.84 |
|  | *Haploporus* sp. LR11231 | 97 | 90.32 |
|  | *H. grandisporus* KE-17-242 | 100 | 89.58 |
|  | *H. grandisporus* KE-17-239 | 100 | 89.58 |
|  | *H. grandisporus* KE-17-282 | 100 | 89.58 |
|  | *H. longisporus* JV 1906/C11-J | 94 | 87.54 |
