# Supplementary material for: ﻿Outline, phylogenetic and divergence times analyses of the genus Haploporus (Polyporales, Basidiomycota): two new species are proposed
Source: MycoKeys. 2023 Jul 24;98:233–52. doi: 10.3897/mycokeys.98.105684 (PMC10390986; doi:10.3897/mycokeys.98.105684)
Supplement: Supplementary material 1 — The supplementary materilas in this study [file mycokeys-98-233-s001.zip › Supplementary Materials/Table S1.docx]

**Table S1.** Specimens and GenBank accession numbers used in this study.

| Species | Specimen | GenBank Accession nos. | |
| --- | --- | --- | --- |
|  |  | ITS | LSU |
| *Agaricus campestris* | LAPAG370 | KM657927 | KR006607 |
| *Amylocorticium cebennense* | CFMR:HHB 2808 | GU187505 | GU187561 |
| *Antrodia tanakae* | Cui 9743 | KR605814 | KR605753 |
| *Aphanobasidium pseudotsugae* | CFMR:HHB 822 | GU187509 | GU187567 |
| *Athelia epiphylla* | CFMR:FP 100564 | GU187501 | GU187558 |
| *Boletopsis leucomelaena* | AFTOL-ID 1527 | DQ484064 | DQ154112 |
| *Boletus edulis* | HMJAU4637 | JN563894 | KF112455 |
| *Callistosporium graminicolor* | AFTOL-ID 978 | DQ484065 | AY745702 |
| *Coltricia perennis* | Cui 10318 | KU360686 | KJ000224 |
| *Dacryopinax spathularia* | AFTOL 454 | AY854070 | AY701525 |
| *Datronia mollis* | RLG6304sp | JN165002 | JN164791 |
| *Datronia stereoides* | Holonen | KC415179 | KC415196 |
| *Favolus acervatus* | Dai 10749b | KX548953 | KX548979 |
| *Favolus niveus* | Cui 11129 | KX548955 | KX548981 |
| *Gloeophyllum sepiarium* | Wilcox-3BB | HM536091 | HM536061 |
| *Jaapia argillacea* | CBS 252.74 | GU187524 | GU187581 |
| *Lactarius deceptivus* | AFTOL-ID 682 | AY854089 | AY631899 |
| *Lentinus arcularius* | Cui 10998 | KX548973 | KX548995 |
| *Lentinus substrictus* | Wei 1582 | KU189767 | KU189798 |
| *Neofavolus alveolaris* | Dai 11290 | KU189768 | KU189799 |
| *Neofavolus mikawai* | Cui 11152 | KU189773 | KU189804 |
| *Neurospora crassa* | OR74A | HQ271348 | AF286411 |
| *Onnia tomentosa* | Dai 22935 | OM677242 | OM677249 |
| *Picipes atratus* | Dai 13375 | KX900042 | KX900158 |
| *Picipes submelanopus* | Dai 13294 | KU189770 | KU189801 |
| *Polyporus auratus* | Dai 13665 | KX900056 | KX900172 |
| *Polyporus umbellatus* | Pen 13513 | KU189772 | KU189803 |
| *Pseudofavolus cucullatus* | Dai 13584A | KX900071 | KX900185 |
| *Sanghuangporus sanghuang* | Cui 14419 | MF772789 | MF772810 |
| *Schizophyllum radiatum* | AFTOL-ID-516 | AY571060 | AY571023 |
| *Ustilago maydis* | AFTOL 505 | AY854090 | AF453938 |
